# Ultrasensitive Negative Feedback Control: A Natural Approach for the Design of Synthetic Controllers

**DOI:** 10.1371/journal.pone.0161605

**Published:** 2016-08-18

**Authors:** Francesco Montefusco, Ozgur E. Akman, Orkun S. Soyer, Declan G. Bates

**Affiliations:** 1 Department of Information Engineering, University of Padova, Padova, Italy; 2 College of Engineering, Mathematics and Physical Sciences, University of Exeter, Exeter, United Kingdom; 3 School of Life Sciences, University of Warwick, Coventry, United Kingdom; 4 School of Engineering, University of Warwick, Coventry, United Kingdom; Imperial College London, UNITED KINGDOM

## Abstract

Many of the most important potential applications of Synthetic Biology will require the ability to design and implement high performance feedback control systems that can accurately regulate the dynamics of multiple molecular species within the cell. Here, we argue that the use of design strategies based on combining ultrasensitive response dynamics with negative feedback represents a natural approach to this problem that fully exploits the strongly nonlinear nature of cellular information processing. We propose that such feedback mechanisms can explain the adaptive responses observed in one of the most widely studied biomolecular feedback systems—the yeast osmoregulatory response network. Based on our analysis of such system, we identify strong links with a well-known branch of mathematical systems theory from the field of Control Engineering, known as Sliding Mode Control. These insights allow us to develop design guidelines that can inform the construction of feedback controllers for synthetic biological systems.

## Introduction

The development of appropriate design frameworks for the construction of synthetic feedback controllers is an important open problem in Synthetic Biology that has recently begun to attract significant attention from the Control Engineering research community [1–3]. A key requirement for any such framework is that it is consistent with the nature of biological information processing, in order that any resulting designs can be readily implemented via biomolecular circuitry. This represents a significant challenge, since (mainly for historical reasons) many of the implicit assumptions underlying control theory are based on consideration of dynamical properties that arise in the context of physical, rather than biological, systems. For example, the assumption that the dynamics of both the system to be controlled and the feedback controller can be well approximated by linear models is widely made in many branches of feedback control theory. This assumption is often valid for many physical systems (from motors to aircraft to power networks) because these systems have been purposefully designed by engineers to provide predominantly linear response dynamics, since this significantly simplifies their analysis and control.

**Fig 1:**
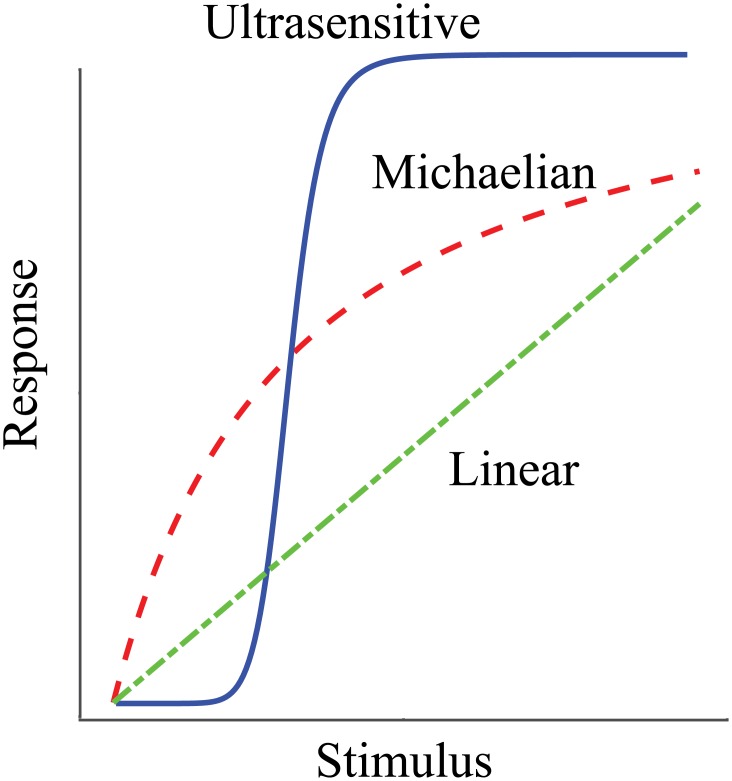
Steady-state input-output characteristics. Relationships for linear, Michaelian and ultrasensitive systems.

This contrasts strongly with the situation in many biological contexts, where evolution often results in systems that display strongly nonlinear dynamics. A prime example of such nonlinear dynamics is represented by the phenomenon of ultrasensitivity, in which the gain of the system (ratio of output signal to input signal) changes from very low, to very high, and then back to very low as the magnitude of the input signal increases. The resulting sigmoidal shape of the system’s response (Fig 1) is a widely observed characteristic of many different biological systems [4, 5], and can be achieved via a variety of different molecular mechanisms, including dimerization of transcription factors [6], use of scaffolding proteins in MAPK systems [7], and branching in bacterial phosphorylation/de-phosphorylation cycles [8]. In addition to their nonlinear dynamics, all processes in nature are noisy. The functional roles of noise are many and diverse [9, 10]: on the one hand, noise can be an undesired property of the process, due to entropy-increasing effects that limit the fidelity and robustness of signaling pathways and, then, it is crucial to control and attenuate it; on the other hand, noise can be a surprising beneficial effect by increasing functional heterogeneity and thus diversity (accelerating, for instance, the pace of evolution) and, then, it becomes important to exploit and amplify it. In the last two decades the effects of feedback (ubiquitous in biology) on noise have been well investigated: two seminal works suggested negative feedback as a mechanism for attenuating the effect of noise [11, 12]. Further theoretical and experimental research has revealed a more intricate relation between negative feedback and noise, indicating that noise can be attenuated or amplified depending on the feedback strength [13–15].

Interestingly, signalling systems implementing phosphorylation-dephosphorylation cycles in concert with negative feedback loops have been shown to exhibit adaptive characteristics, i.e. an initial response to a persistent external stimulus eventually returns to its pre-stimulus level [16–20]. This adaptive capability (referred to as “disturbance rejection” in control theory) is a key requirement for many feedback control systems, since it allows the system to robustly maintain specified levels of performance despite the inevitable presence of environmental fluctuations and disturbances.

In the following, we show that a control model that combines ultrasensitive responses with negative feedback, resulting in a control model we call ultrasensitive negative feedback (*U*_*NF*_), provides a plausible explanation for the adaptive responses observed in one of the most widely studied biomolecular feedback systems—the yeast osmoregulatory response network [21–27]. Indeed, the yeast osmoregulation system implements the archetypical mitogen-activated protein kinase (MAPK) pathway, as well as a two-component signaling system, both of which control downstream gene expression. Both the two-component and MAPK cascades have been shown theoretically and experimentally to embed ultrasensitive dynamics [4, 5, 28–31], while gene expression dynamics are usually implemented as a Hill function (e.g. see [27]). Moreover, the presence of ultrasensitivity has also been suggested for the Fps1 glycerol channels determining the glycerol export rate [25, 27]. Thus, the presence of ultrasensitivity in the yeast osmoregulation system is broadly accepted. Therefore, these findings point to the possibility of *U*_*NF*_ based mechanisms that allow yeast to achieve adaptive responses. Subsequent analysis of such controller models reveals strong links with a particular class of nonlinear feedback controllers, known as Sliding Mode controllers, whose performance and robustness properties are well known to engineers [32–34]. Based on these insights, we develop design guidelines that could be exploited by Synthetic Biologists to inform the design of synthetic feedback control circuits for a wide variety of potential applications.

## Methods

### Osmoregulation as a feedback control system

**Fig 2:**
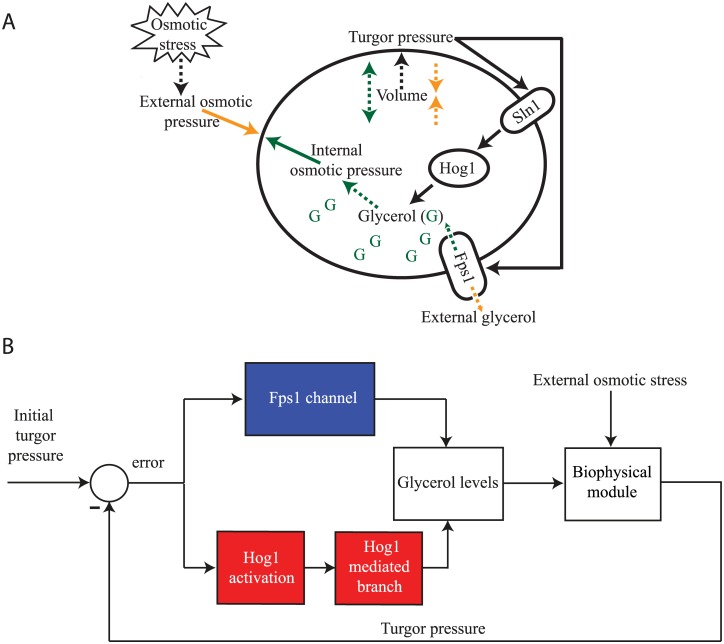
Representations of the yeast osmosensing system. (A) Schematic depiction of the osmosensing response. After an osmotic stress, the external osmotic pressure increases and water diffuses out of the cell, causing the turgor pressure and volume to decrease. Two parallel control paths are activated to regain volume and turgor pressure by adjusting the glycerol production: the activation of the Hog1 protein and all the corresponding mechanisms that promote glycerol production; the Fps1 channel, which regulates the outflow of glycerol and is immediately closed after the shock. (B) Engineering block diagram representation of a control model for the osmoregulation system.

The osmoregulation system can be naturally abstracted as a feedback control system comprised of two separate mechanisms that act to adjust glycerol production in order to keep the cell’s turgor pressure and volume constant in the face of environmental changes (Fig 2): 1) the regulation of the membrane protein Fps1 determining the glycerol export rate (the Fps1 channel, blue box of Fig 2B); 2) the activation of the high osmolarity glycerol (HOG) mitogen-activated protein kinase (MAPK) signalling and the corresponding Hog1-dependent mechanisms that promote glycerol production (the series of the Hog1 activation system and the Hog1 mediated branch, red boxes of Fig 2B). Our model therefore consists of three main compartments: 1) a biophysical module describing how the cell volume and the turgor pressure are affected by varying extra–cellular osmolarity; 2) a control system comprised of two parallel mechanisms that determines the glycerol levels; 3) a glycerol module that determines the intra- and extra-cellular glycerol concentration and the corresponding biophysical properties of the system. The mathematical representations employed for each of these modules are described in the following sections.

#### The biophysical module

The biophysical model is based on the work presented in [22]. The system is modelled by considering the dependencies between cell volume *V*, the turgor pressure *P*_*t*_, the intra–cellular osmotic pressure *P*_*i*_ and the extra–cellular osmotic pressure *P*_*e*_. At any given time *t*, *P*_*i*_(*t*), *P*_*e*_(*t*) and *P*_*t*_(*t*) determine the flow of water across the cell membrane, which is proportional to (*P*_*i*_(*t*) − *P*_*e*_(*t*) − *P*_*t*_(*t*)). Assuming that the cell volume is only affected by the inflow and outflow of water, then the change in volume can be expressed as

$$\begin{array}{c} \frac{dV}{dt}=k_{p1}\left(P_{i}\left(t\right)-P_{e}\left(t\right)-P_{t}\left(t\right)\right)\;, \end{array}$$ (1)

with *k*_*p*1_ denoting a hydraulic water permeability constant. At equilibrium (equil.), i.e. constant volume and no net flow of water over the membrane, Eq (1) reduces to

$$\begin{array}{c} P_{i}=P_{e}+P_{t}\;.\;\;\;\;\;\left(equil.\right) \end{array}$$

The only osmolyte considered explicitly in the model is glycerol (*Gly*); hence, ions and other small molecules that change upon osmotic shock [35] are not considered. This assumption is motivated by experimental results from [36], where the authors found that glycerol counter-balances approximately 80% of applied NaCl in *S. cerevisiae*. Therefore, the intra-cellular osmotic pressure, according to van’t Hoff’s law, is expressed as

$$\begin{array}{c} P_{i}\left(t\right)=\frac{s+Gly(t)}{V\left(t\right)-V_{b}}\;, \end{array}$$ (2)

with *s* being the concentration of the sum of osmolytes (assumed constant) other than glycerol present in the cell, and *V*_*b*_ being the non-osmotic volume of the cell, subsuming non-polar cellular components, such as membranes. According to Eq (2), the intra–cellular osmotic pressure increases with the glycerol concentration, which can be used to control the turgor pressure of the cell.

The extra-cellular osmotic pressure is only modified by the input signal, *u*(*t*), for example the applied salt stress, and is then independent of changes in other variables. Hence

$$\begin{array}{c} P_{e}\left(t\right)=P_{e_{equil}}+u\left(t\right)\;, \end{array}$$ (3)

where *P*_*e*_*equil*__ is the extra-cellular osmotic pressure at equilibrium (at *t* = 0, *P*_*e*_*equil*__ = *P*_*e*_(0) = *P*_*i*_(0) − *P*_*t*_(0)).

The turgor pressure is linearly dependent on the volume according to [37], in the following manner:

$$\begin{array}{c} P_{t}\left(t\right)=\epsilon \left(\frac{V(t)}{V(0)}-1\right)+P_{t}\left(0\right)\;. \end{array}$$ (4)

Here, *V*(0) is the initial volume, *P*_*t*_(0) is the initial turgor pressure, and *ϵ* is the volumetric elastic modulus. By expressing the volume at which *P*_*t*_ = 0 with the notation *V*^*P*_*t*_ = 0^, Eq (4) can be rewritten as

$$\begin{array}{c} P_{t}\left(t\right)=\left\{\begin{array}{cc} P_{t}\left(0\right)\frac{V\left(t\right)-V^{P_{t}=0}}{V\left(0\right)-V^{P_{t}=0}}\;, & V\left(t\right)>V^{P_{t}=0}\;,\\ 0\;, & \text{otherwise}\;. \end{array}\right. \end{array}$$ (5)

#### The controller modules

There are two branches of control in the model: the first represents the closure of Fps1 glycerol transporter channels as a reaction to osmotic shock, causing accumulation of glycerol and an increase in the intra-cellular osmotic pressure *P*_*i*_ [38]; the second the activation of the Hog1 protein and the corresponding Hog1-dependent mechanisms that promote glycerol production (such as the transcriptional activation of genes that encode enzymes involved in glycerol production and potential protein-protein interactions initiated by Hog1 in the cytoplasm or nucleus that lead to glycerol accumulation) [21, 24, 25].

The input signal, the error *e*(*t*), arriving at the two control branches, defined by

$$\begin{array}{c} e\left(t\right)=P_{t}\left(0\right)-P_{t}\left(t\right) \end{array}$$ (6)

is the difference between the initial and current turgor pressure.

The output of the Fps1 branch, *u*_*Fps*1_(*t*), which corresponds to the response of the transporter channels, is given by

$$\begin{array}{c} u_{Fps1}\left(t\right)=k_{Fps1}-\text{sgn}\left(e\left(t\right)\right)k_{Fps1}\cdot \frac{{\left|e\left(t\right)\right|}^{n_{Fps1}}}{\beta_{Fps1}{\left|e\left(t\right)\right|}^{n_{Fps1}}+K_{Fps1}}\;, \end{array}$$ (7)

where *β*_*Fps*1_ = 1 − exp^*k*_*e*_*Fps*1__(1 − *n*_*Fps*1_)^, *K*_*Fps*1_ = *P*_*t*_(0)exp^*k*_*e*_*Fps*1__(1 − *n*_*Fps*1_)^, *k*_*e*_*Fps*1__ is a constant and *n*_*Fps*1_ is the exponent of the Hill function that determines the dynamics of the Fps1 controller. The function *u*_*Fps*1_ returns real values in the interval [0, *k*_*Fps*1_], where 0 corresponds to completely closed and where *k*_*Fps*1_ is the glycerol permeability coefficient in a completely open Fps1 channel. Note that we use the sign and absolute value of the error to allow the controller to work in a symmetrical way for positive and negative values of the error.

To describe the dynamics of the Hog1 activation we use the following first order linear system, as done in [24]:

$$\begin{array}{c} {\dot{u}}_{HOG}\left(t\right)=b_{HOG}e\left(t\right)-a_{HOG}u_{HOG}\left(t\right) \end{array}$$ (8)

Here *b*_*HOG*_ and *a*_*HOG*_ are constants, and *u*_*HOG*_ represents the activation of Hog1. We constrain *b*_*HOG*_ and *a*_*HOG*_ to assume similar values, to achieve linear dynamics between the error and the Hog1 activity as proposed by [24] based on which components of the system display adaptive dynamics.

To model the Hog1 mediated feedback control branch, using an *U*_*NF*_ controller, we implement a Hill-type function so that the output of this controller is given by

$$\begin{array}{ccc} v_{HOG}\left(t\right) & = & \left\{\begin{array}{cc} k_{HOG}\frac{u_{HOG}{\left(t\right)}^{n_{HOG}}}{\beta_{HOG}u_{HOG}{\left(t\right)}^{n_{HOG}}+K_{HOG}}\;, & u_{HOG}\left(t\right)>0\;,\\ 0\;, & \text{otherwise}\;, \end{array}\right. \end{array}$$ (9)

where *k*_*HOG*_ is the gain of the controller, *β*_*HOG*_ = 1 − exp^*k*_*e*_*HOG*__(1 − *n*_*HOG*_)^, *K*_*HOG*_ = exp^*k*_*e*_*HOG*__(1 − *n*_*HOG*_)^ and *k*_*e*_*HOG*__ and *n*_*HOG*_ are constants. Note that the Hog1 mediated controller only works for positive values of the input and is switched off for negative values.

Eqs (7) and (9) allow the controllers to evolve from proportional (*n*_*Fps*1_ = *n*_*HOG*_ = 1) to ultrasensitive dynamics (*n*_*Fps*1_, *n*_*HOG*_ > 1). Indeed, for *n*_*Fps*1_ = *n*_*HOG*_ = 1, the parameters *β*_*Fps*1_ = 1 − exp^*k*_*e*_*Fps*1__(1 − *n*_*Fps*1_)^ and *β*_*HOG*_ = 1 − exp^*k*_*e*_*HOG*__(1 − *n*_*HOG*_)^ become 0, *K*_*Fps*1_ = *P*_*t*_(0)exp^*k*_*e*_*Fps*1__(1 − *n*_*Fps*1_)^ = *P*_*t*_(0) and *K*_*HOG*_ = exp^*k*_*e*_*HOG*__(1 − *n*_*HOG*_)^ = 1, and thus we end up with the following proportional (*P*_*NF*_) controllers:

$$\begin{array}{ccc} u_{Fps1}\left(t\right) & = & \left\{\begin{array}{cc} k_{Fps1}\frac{P_{t}\left(0\right)-e\left(t\right)}{P_{t}\left(0\right)}\;, & e(t)\geq 0\;,\\ k_{Fps1}\frac{P_{t}\left(0\right)+e\left(t\right)}{P_{t}\left(0\right)}\;, & e(t)<0\;, \end{array}\right. \end{array}$$ (10)

$$\begin{array}{ccc} v_{HOG}\left(t\right) & = & \left\{\begin{array}{cc} k_{HOG}\cdot u_{HOG}\left(t\right)\;, & u_{HOG}\left(t\right)>0\;,\\ 0\;, & \text{otherwise}\;. \end{array}\right. \end{array}$$ (11)

Finally, we consider the case where the Hog1 pathway implements an integrator as proposed in [24], where the authors hypothesised that, to achieve perfect adaptation, the system implements an integral feedback via a non-transcriptional pathway that requires the Hog1 activity. Then, the output of this channel is described by the following equation:

$$\begin{array}{c} v_{HOG}\left(t\right)=k_{HOG}\cdot \int_{t-T_{m}}^{t}u_{HOG}\left(\tau \right)d\tau \;, \end{array}$$ (12)

where *k*_*HOG*_ is the gain of the channel and *T*_*m*_ is the time window of the integral. In the case of infinite integration time (*T*_*m*_ = ∞), the controller implements an ideal integrator (*I*_*NF*_). *I*_*NF*_ takes into account the complete history of the process and produces an output value proportional to the integral of the error (over a potentially infinite integration period). If *T*_*m*_ is finite, the controller implements a finite integrator (*FI*_*NF*_), which is able to store only a limited history of the error.

#### The glycerol module

The exchange of internal and external glycerol, *u*_*Diff*_, over the Fps1 channel is modelled using Fick’s first law of diffusion as

$$\begin{array}{c} u_{Diff}\left(t\right)=u_{Fps1}\left(t\right)\left(\frac{Gly(t)}{V\left(t\right)-V_{b}}-\frac{Gly_{e}\left(t\right)}{V_{e}}\right), \end{array}$$ (13)

with *V*_*e*_ being the extra-cellular volume, *Gly* being the intra-cellular glycerol and *Gly*_*e*_ being the glycerol in the extra-cellular compartment. The extra-cellular glycerol, depending only on the diffusion over the Fps1 channel, is described by

$$\begin{array}{c} \frac{dGly_{e}}{dt}=u_{Diff}\left(t\right)\;. \end{array}$$ (14)

Intra-cellular glycerol production, which is used to control the turgor pressure of the cell by changing the intra-cellular osmotic pressure (see Eq (2)), is expressed by combining the output of the two controllers described above:

$$\begin{array}{c} \frac{dGly}{dt}=v_{HOG}\left(t\right)-u_{Diff}\left(t\right)\;. \end{array}$$ (15)

Our model contains 20 parameters as reported in S1 Table. However, four of these are dependent parameters which do not need to be constrained.

### Optimization of the parameters for different control schemes against experimental datasets

For each control scheme we use global optimization algorithms to optimize the model parameters to fit the available experimental data presented in [24], in particular the volume and the Hog1 responses to step shocks of 0.2, 0.4 and 0.6 M of NaCl. S2 Table reports the optimal model parameters obtained for each combination of dataset and control scheme. Optimisation problems were formulated by the square sum of the errors between the simulated responses (volume and Hog1) produced by the model to different osmotic stresses and the experimental data as follows:

$$\begin{array}{c} \min_{p}\;J=\sum_{j}\sum_{i}(V_{j}\left(t_{i}\right)-{\hat{V}}_{j}{\left(t_{i},p)\right)}^{2}+(u_{HOGj}\left(t_{i}\right)-{\hat{u}}_{HOGj}{\left(t_{i},p)\right)}^{2}, \end{array}$$ (16)

where *p* is the set of model parameters, *V*_*j*_(*t*_*i*_) and *u*_*HOGj*_(*t*_*i*_) are the experimental volume and Hog1 measurements, respectively, at time *t*_*i*_ for the *j*-th experiment (step shock with different amplitude) and ${\hat{V}}_{j}\left(t_{i}\right)$ and ${\hat{u}}_{HOGj}\left(t_{i},p\right)$ are the volume and Hog1 responses of the model, respectively, at time *t*_*i*_ for the *j*-th experiment.

Note that in our model we do not consider any growth mechanism; therefore, when the volume is completely recovered, i.e. *V*_*j*_(*t*_*i*_) > 1, the data points are assumed equal to 1 for our computations, as in [27]. For each control scheme, we optimize the control parameters and the main biophysical parameters (which are $V^{P_{t}}=0$, defining the volume at which the turgor pressure is zero, and *k*_*p*1_, the water permeability coefficient). All the other parameters, which showed little effect when varied, are fixed as reported in S1 Table.

For the optimization, we use a hybrid Genetic Algorithm (GA) [39], that combines the most well-known type of evolutionary algorithm with a local gradient-based algorithm [40, 41] to ensure the computation of globally optimal solutions. Indeed, most optimization problems encountered in biology involve non-convex search spaces and thus any local optimization algorithm, which uses gradient information of the cost function to find the search direction for determining the optimum, may only provide a local, rather than a global solution, depending on where in the search space the optimization starts. GA, in contrast, uses a heuristic search technique that mimics the process of natural selection and then requires only the calculation of the cost function. Therefore, due its stochastic nature, GA can be expected to have a much better chance of converging to a global optimum than a local optimization algorithm, and a hybrid GA makes the solution more robust. For the computation, we use the function *ga* from the MATLAB Global Optimization Toolbox [42] and *fmincon* from the MATLAB Optimization Toolbox [43], as the local algorithm. We repeat the hybrid GA algorithm five times and select the parameter set that gives the optimal value of the cost function *J*.

### Availability of models and computer code

MATLAB code containing the files for generating the results presented in the main text and Supporting Information is provided as an additional S1 File.

## Results

### A model based on *U*_*NF*_ control explains experimental data on yeast osmoregulation

We focus on yeast osmoregulation as a model system to investigate the possible mechanisms for adaptive response dynamics. Several experimental studies have provided detailed data on the dynamics of the yeast osmoshock response, and have elucidated the molecular pathways involved [21, 24, 27]. In brief, yeast perceives a change in external osmolyte conditions (e.g. salt shock) as a drop in cell volume and turgor pressure, sensed through its membrane bound osmosensor, SLN1 (see Fig 2A).

**Fig 3:**
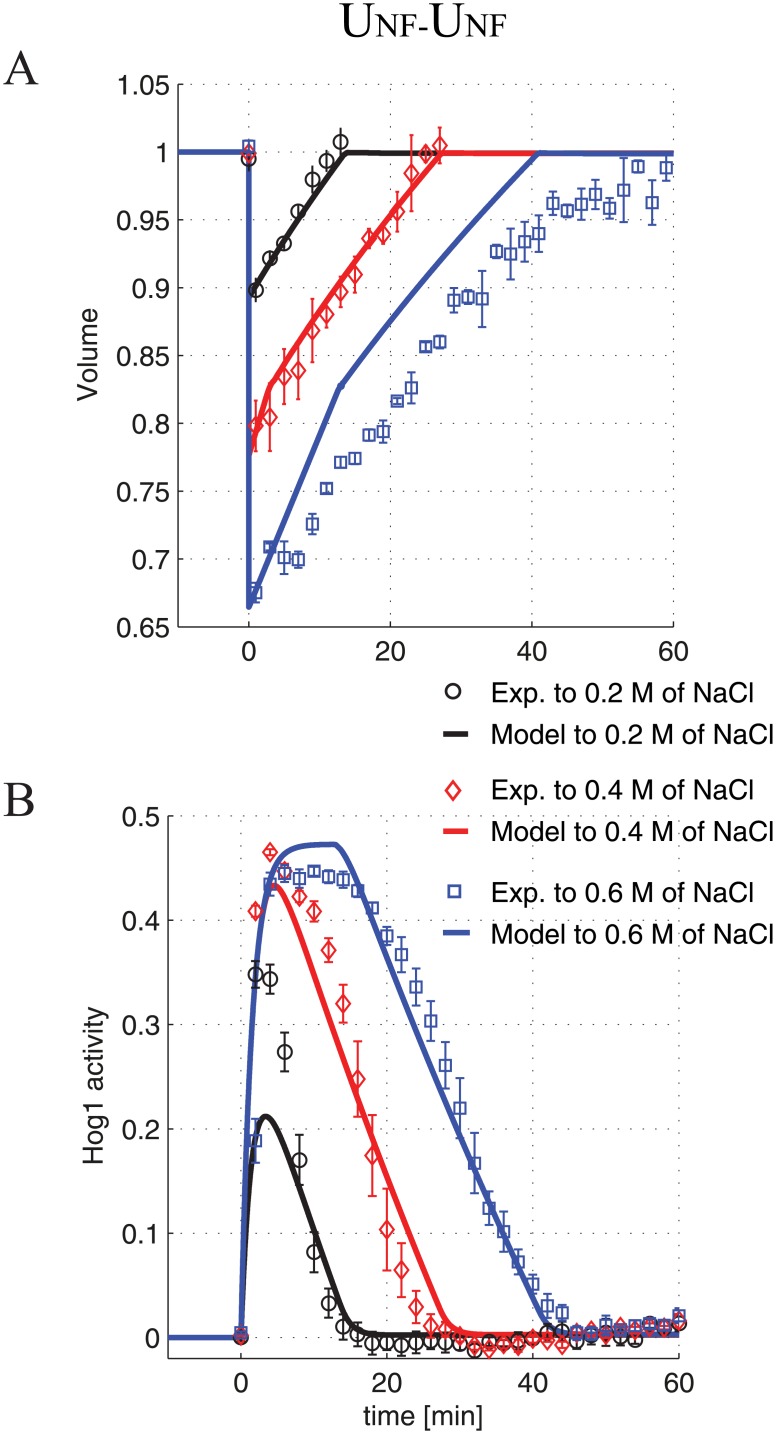
Best fit to osmoshocks for the *U*_*NF*_-*U*_*NF*_ model. Best fit to the experimental dataset for the cell volume (A) and the Hog1 (B) responses to three step osmoshocks of different magnitude; the experimental data for 0.2, 0.4, and 0.6 M of NaCl are indicated by black circles, red diamonds, and blue squares, respectively. The corresponding coloured solid lines represent the optimised model responses.

SLN1 is part of a two-component signalling cascade that leads to activation of a mitogen-activated protein kinase (MAPK) cascade, which leads to the phosphorylation of the transcription factor Hog1 [21]. Phosphorylated Hog1 translocates to the nucleus and activates the expression of genes encoding enzymes involved in glycerol production. In addition, a drop in turgor pressure leads to the closing of the membrane bound glycerol channel Fps1. The resulting accumulation of glycerol inside the cell reinstates the turgor pressure and cell volume, and thus underpins the observed adaptive dynamics in cell volume. We model the described osmoregulatory network as a feedback control system, where the SLN1 receptor is seen as computing the difference (i.e. error) between the current and an ideal turgor pressure (where the latter corresponds to the steady state cell volume), while the Fps1 channel and the Hog1 pathway leading to glycerol activation are seen as feedback controllers that process the error and feed their response back to the system (see Fig 2B and Methods). The system to be controlled is considered to involve the cellular glycerol levels and their effect on turgor pressure and volume (see Methods). This model architecture allows us to investigate the effect of implementing different types of response dynamics for the two feedback controllers on the model’s ability to match the available experimental data on responses to osmotic shocks. Within the yeast osmoregulation system, the presence of ultrasensitivity has been either demonstrated or suggested in several parts of the system, e.g. the SLN1 two-component system preceding the MAPK cascade [44], the MAPK cascade terminating at Hog1 [5], the Fps1 glycerol channels [25, 27] and the mechanisms mediated by Hog1 that promote glycerol accumulation, such as the transcriptional activation of genes encoding enzymes involved in glycerol production and potential protein-protein interactions initiated by Hog1 in the cytoplasm or nucleus that lead to glycerol accumulation [24, 25]. To capture these observations, we develop a model that implements an *U*_*NF*_ controller for both the Fps1 and Hog1 mediated feedback systems, named *U*_*NF*_-*U*_*NF*_ (Methods, Eqs (7) and (9)). Optimising the parameters of this model within biologically feasible ranges produces an excellent fit to datasets on yeast responses to stepwise osmotic shocks of various sizes (Fig 3 and Methods).

For the purposes of comparison, two canonical linear controllers, proportional negative feedback (*P*_*NF*_) and integral negative feedback (*I*_*NF*_), are also implemented in the model and optimised against the same datasets (see Methods and S1 Appendix for the main properties of these controllers using a generic control scheme). Table 1 reports all the possible control schemes here exploited for explaining the experimental data.

**Table 1:** Different control schemes exploited for reproducing experimentally observed responses of yeast to different levels of osmoshock.

Control Models	Fps1 channel	Hog1 mediated branch
**P**_**NF**_-**P**_**NF**_	Proportional negative feedback (*P*_*NF*_)	Proportional negative feedback (*P*_*NF*_)
**P**_**NF**_-**I**_**NF**_	Proportional negative feedback (*P*_*NF*_)	Integral negative feedback (*I*_*NF*_)
**P**_**NF**_-**F** **I**_**NF**_	Proportional negative feedback (*P*_*NF*_)	Finite integral negative feedback (*FI*_*NF*_)
**U**_**NF**_-**I**_**NF**_	Ultrasensitive negative feedback (*U*_*NF*_)	Integral negative feedback (*I*_*NF*_)
**U**_**NF**_-**F** **I**_**NF**_	Ultrasensitive negative feedback (*U*_*NF*_)	Finite integral negative feedback (*FI*_*NF*_)
**U**_**NF**_-**U**_**NF**_	Ultrasensitive negative feedback (*U*_*NF*_)	Ultrasensitive negative feedback (*U*_*NF*_)

The *P*_*NF*_ controller is commonly used in control engineering and simply amplifies the error by a certain linear gain factor. Thus, *P*_*NF*_ control in itself is not expected to result in adaptive response dynamics, unless the process it controls (in this case, the biophysical model of glycerol accumulation and its effect on volume) implement an *I*_*NF*_, required for achieving adaptation as shown through analytical results from control theory [45–48]. In particular, to see if *P*_*NF*_ control alone can explain the observed data, we develop a model (named *P*_*NF*_-*P*_*NF*_), which assumes that both the Hog1 and Fps1 mediated feedback controllers implement a *P*_*NF*_ control (Methods, Eqs (10) and (11)). By optimizing the model parameters so as to get the best possible fit to the experimental data (see Methods), we find that this model provides a very poor fit, as shown in S1 Fig. In particular, the large steady-state errors produced by this model, and the lack of adaptation, clearly indicate that the controlled process itself does not contain an integrator. Stated biologically, the dynamics of glycerol accumulation itself and its connection to turgor pressure and volume cannot, on their own, explain the observed data. Then, we investigate the performance of a model implementing an *I*_*NF*_ controller. Based on the hypothesis that the observed adaptive dynamics must require an *I*_*NF*_ controller, a previous study placed this type of controller on the Hog1 mediated feedback route [24]. This conclusion was based on the fact that the elements in a control system placed before an *I*_*NF*_ controller must show adaptive dynamics, whilst those placed after such a controller will not. In the case of the yeast osmoregulation system, glycerol levels in the yeast osmoregulation do not adapt, while Hog1 levels do, and thus leading to the proposal that the integral controller resides in the Hog1 mediated feedback path [24]. Therefore, we develop a model (named *P*_*NF*_-*I*_*NF*_) implementing a *P*_*NF*_ controller for the Fps1 channel and an *I*_*NF*_ controller for the Hog1 mediated branch. We find that the *P*_*NF*_-*I*_*NF*_ can achieve a good fit to all experimental data, when optimized (see S2 Fig). However, not all responses to different osmoshock strengths are equally well captured with such a model. We find that achieving a better fit to high levels of osmoshock reduced the fit to low levels and *vice versa*. In particular, a good fit to low (high) levels of osmoshock requires the integral control gain to be optimized to high (low) values (see S3 Fig). Increasing the gain of the integrator to achieve a faster response and improve the fitting to the experimental data for low levels of osmoshock (such as the stress input of 0.2 M of NaCl), however, results in a worse fitting to data for higher levels of osmoshock and overshoot in the volume response, which is not observed experimentally (see panel A in S3 Fig). When we consider an integral controller with a more biologically plausible finite integration window, *T*_*m*_ (i.e. shorter memory, termed *FI*_*NF*_), the difference between simulated and experimental data for such a model (named *P*_*NF*_-*FI*_*NF*_) becomes greater than that achieved by *P*_*NF*_-*I*_*NF*_ and, in particular, the fit to adaptation levels is much worse (see S4 Fig). We also develop a model implementing *U*_*NF*_ controller for the Fps1 feedback channel and an *I*_*NF*_ or *FI*_*NF*_ controller for the Hog1 mediated feedback branch, named *U*_*NF*_-*I*_*NF*_ and *U*_*NF*_-*FI*_*NF*_, respectively (see S5 and S6 Figs). In all cases, however, the *U*_*NF*_-*U*_*NF*_ model is seen to exhibit a significantly better match to the available experimental data. S2 Table reports the *J* values calculated using Eq (16), showing the corresponding model scores: a lower value of *J* indicates a better fit to the data, and *U*_*NF*_-*U*_*NF*_ gets the lowest value. However, the loss function does not take into account the different number of parameters for each model without thereby penalizing models with a larger number of parameters (as *U*_*NF*_-*U*_*NF*_). Therefore, we also compute other standard scores as Akaike information criterion (AIC) [49], Bayesian information criterion (BIC) [50] and Akaike’s final prediction-error criterion (FPE) [51]. In general BIC tends to penalize complex models more heavily, on the other hand AIC and FPE tend to choose models which are too complex as the number of data goes to infinity. For all the scores (whose values are reported in S2 Table), the *U*_*NF*_-*U*_*NF*_ model achieves the lowest values, further confirming that it is the model that is best capable of reproducing the multiple experimental datasets.

Note that our model, in line with all previous models [21–27], is based on the assumption of deterministic dynamics. Indeed, experimental studies have indicated that the noise levels in the system are low due to the abundance of Hog1 [24, 52–54], and then a deterministic model is well-suited for describing the osmoregulation system dynamics. However, we also investigate the effects of noise on controller performance for the *U*_*NF*_-*U*_*NF*_ model, by adding normally distributed noise to the outputs of the two controllers (see S7 Fig): the results are in line with those obtained without noise (see Fig 3), the responses for the different inputs are robust to the effects of noise as the deviation between the simulated and experimental data is limited, and we can state that the deterministic solution of the *U*_*NF*_-*U*_*NF*_ model represents the mean of stochastic simulations.

### *U*_*NF*_ control provides a metabolically efficient means of achieving adaptation

**Fig 4:**
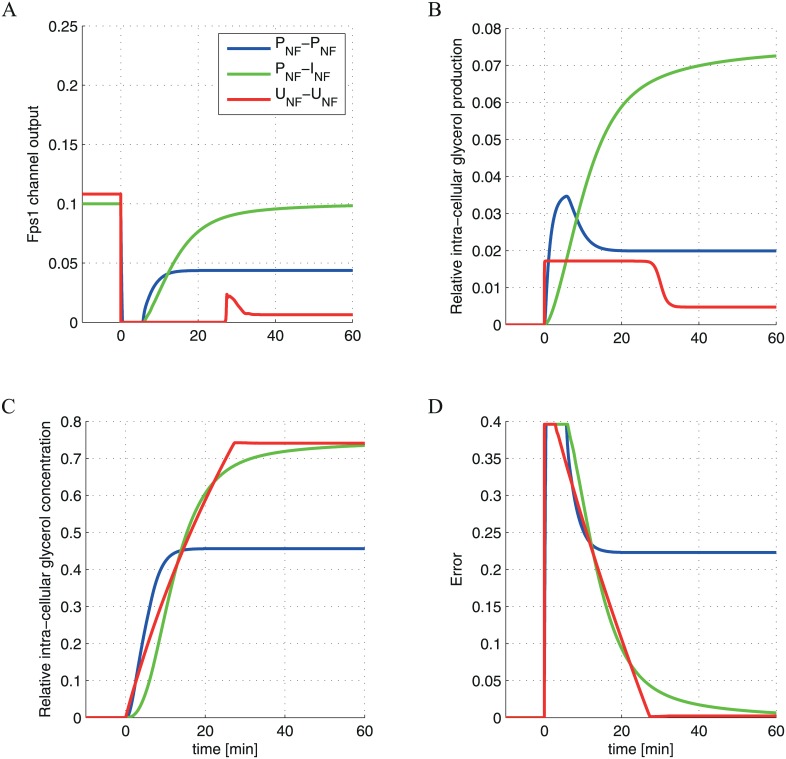
Temporal dynamics of the controllers for the different models. The output of the Fps1 channel (A), the glycerol production (B), the glycerol concentration (C), and the error (D) are shown for the different control schemes (*P*_*NF*_-*P*_*NF*_, *P*_*NF*_-*I*_*NF*_, *U*_*NF*_-*U*_*NF*_ as indicated in the legend), assuming an osmotic stress of 0.4 M of NaCl.

To better understand the basis of the different degrees to which the *P*_*NF*_, *I*_*NF*_ and *U*_*NF*_ control schemes are able to fit the data, we analyze the temporal outputs of different models (Fig 4). As expected, each control scheme responds to the osmoshock by decreasing the Fps1 controller activity (corresponding to a closing of the Fps1 channel) and increasing the glycerol production mediated by the Hog1 controller (Fig 4A–4C). As glycerol accumulates and volume recovers, the error starts to decrease (Fig 4D) resulting in a decrease in the glycerol production mediated by the Hog1 controller (Fig 4B) and the re-opening of the Fps1 channel (Fig 4A). This eventually leads to the system reaching a new dynamical steady state. We find that a crucial aspect of each control scheme’s ability to capture the adaptation dynamics is the interplay between error-mediated changes in the glycerol production and export (i.e. Hog1 vs. Fps1 controller dynamics). When both the controllers incorporate linear dynamics (e.g. for the *P*_*NF*_-*P*_*NF*_ and *P*_*NF*_-*I*_*NF*_ models, where the Fps1 channel implements *P*_*NF*_ and the Hog1 mediated branch implements *P*_*NF*_ or *I*_*NF*_ as proposed in [24], respectively), the Fps1 channel opens before the volume is completely recovered (Fig 4A, blue and green lines), triggering the leakage of glycerol out of the cell prematurely. Thus, higher Hog1 mediated glycerol production is required to increase the glycerol level and recover the cell volume. When both controllers implement *U*_*NF*_, however, the strongly nonlinear controllers respond rapidly to the stress by immediately increasing the glycerol production (Fig 4B, red line) and the Fps1 channel remains partially closed until the error becomes less than a certain threshold.

### *U*_*NF*_ controllers implement a quasi sliding mode control scheme

**Fig 5:**
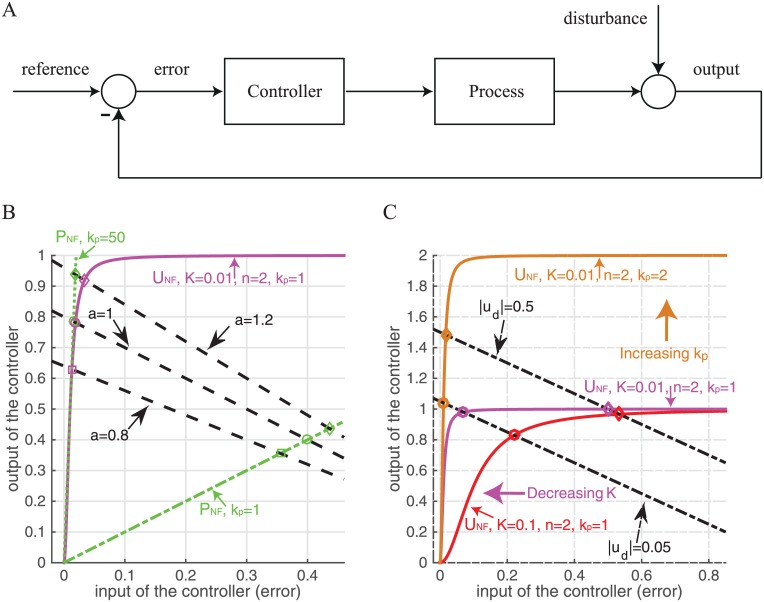
Performance of the generic closed-loop feedback system. (A) Block diagram representation of the generic control system model. (B) Steady state error for the generic closed-loop system, while varying the parameter *a* of the process, with the process parameter *b*, reference signal *r* and step disturbance amplitude *a*_*u*_*d*__ held constant (*b* = *r* = 1, *a*_*u*_*d*__ = 0.2). Intersections of the magenta line with the black dashed straight lines give steady state error values for the *U*_*NF*_ controller (*K* = 0.01, *n* = 2 and *k*_*p*_ = 1). Intersections of the green lines with the black dashed straight lines give steady state error values for the *P*_*NF*_ controller (dashed-dotted line for *k*_*p*_ = 1; dotted line for *k*_*p*_ = 50). (C) Steady state error for the closed-loop system, while varying the amplitude *a*_*u*_*d*__ of the step disturbance, *u*_*d*_, with different values of *k*_*p*_ (red and magenta lines for *k*_*p*_ = 1; orange line for *k*_*p*_ = 2) and *K* (red line for *K* = 0.1; magenta and orange lines for *K* = 0.01) for the *U*_*NF*_ controller (*n* is fixed at 2).

To better understand the ability of ultrasensitive negative feedback to produce adaptive dynamics, and to develop some general guidelines for the design of synthetic controllers based on this approach, we analyse the performance of such a controller using a generic closed-loop feedback control model (see Fig 5). In this simplified system, the controller is employed to maintain a given process at a particular set-point when it is subject to a step disturbance (Fig 5A). The input of the controller is the error signal, *e*, defined as the difference between the desired output, *r* (called the reference signal), and the actual output of the system, *y*. Based on the error signal, the controller manipulates the input to the process, *u*, to reduce the effect of the disturbance, *u*_*d*_, on the output *y* and therefore obtain the desired response. We also analyze the performance of individual *P*_*NF*_, *I*_*NF*_, *FI*_*NF*_ using the generic closed-loop feedback control model and derive analytical expressions for the controller output (see S1 Appendix and S8 Fig). Inline with control theory, we show that for the *P*_*NF*_ controller, achieving an error value close to zero requires a very large gain resulting in very high output signals from the controller even for a very small disturbance (see panel A in S8 Fig). For the *I*_*NF*_ controller, the output of the process is equal to the reference signal at steady state given any step disturbance, i.e. the error is zero at steady state and the system achieves perfect adaptation (see panel B in S8 Fig). However, the time to reach the steady state varies with controller parameters, and making the integration time window finite (*FI*_*NF*_) can destroy the ability of the integral controller to recover the original steady state value (see panels C–E in S8 Fig)).

An ultrasensitive controller (*U*_*NF*_), where the error is processed through a system with ultrasensitive (sigmoidal dose-response) dynamics, before being fed into the process, can be modelled using a simple Hill-type function, so that the input from the controller back to the process can be represented by

$$\begin{array}{c} u\left(t\right)=\text{sgn}\left(e\left(t\right)\right)k_{p}\cdot \frac{{\left|e\left(t\right)\right|}^{n}}{{\left|e\left(t\right)\right|}^{n}+K^{n}}\;. \end{array}$$ (17)

Note that we use the sign and absolute value of the error to allow the controller to work in a symmetrical way for positive and negative values of the error.

For such a controller, an analytical expression for the system output cannot be derived. However, it is possible to show that the *U*_*NF*_ controller can achieve a steady state error value very close to zero. Indeed the steady state error can be visulaized by plotting both sides of Eq (A25) in S1 Appendix as shown in Fig 5B and 5C: the intersections of the straight line *a*(*r* − *e* − *a*_*u*_*d*__) with $\text{sgn}\left(e\right)bk_{p}\cdot \frac{{\left|e\right|}^{n}}{{\left|e\right|}^{n}+K^{n}}$, for different values of *K* and *k*_*p*_, by varying *a*_*u*_*d*__ (Fig 5C), correspond to the steady-states of the system. For the purposes of comparison, Fig 5B also shows the corresponding steady-states obtained with a proportional controller—these are given by the intersection of the straight lines *a*(*r* − *e* − *a*_*u*_*d*__) (dashed black line) and *bk*_*p*_ *e* (dash-dot green line). Note that for any value of *n*, given a small value of *K* and without increasing the gain *k*_*p*_, the ultrasensitive controller can achieve a steady-state error value very close to zero, therefore, high values of *n* and *k*_*p*_ are not needed. In contrast, achieving similarly low levels of error with a proportional controller (dashed green line) would require increasing the gain towards infinity (a requirement that is never practically feasible). Moreover, *U*_*NF*_ provides a more tunable system, where both the maximal response and the point of high sensitivity can be adjusted by changing independent parameters (Fig 5C).

**Fig 6:**
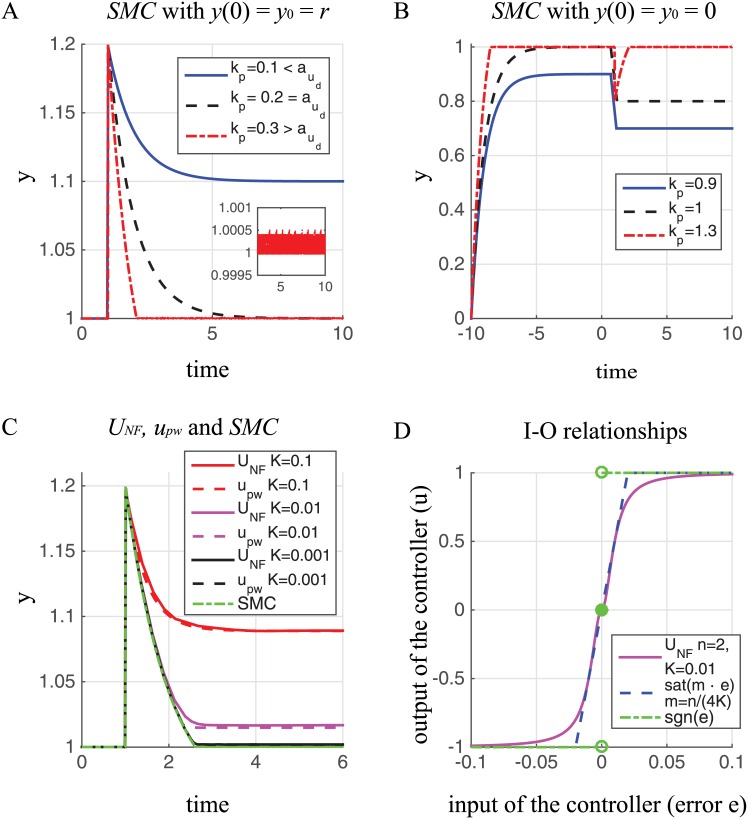
Performance of the generic closed-loop feedback system using *SMC*, *U*_*NF*_, and its approximation by the piecewise function *u*_*pw*_. (A-C) Response dynamics obtained using: (A-B) *SMC*, a sliding mode controller (*u*(*t*) = *k*_*p*_sgn(*e*)) for different values of the gain *k*_*p*_ and *y*(0) = *y*_0_; (C) *U*_*NF*_, its approximation by the piecewise function *u*_*pw*_ and the ideal *SMC*, with *k*_*p*_ = 0.25, *n* = 2 and different values of *K*. The parameters *a*, *b* and the constant reference signal *r* are set equal to 1. The system output is initially equal to the desired constant reference value *y*(0) = *y*_0_ = *r* (A and C), whereas *y*(0) = *y*_0_ = 0 (B). A step disturbance, *u*_*d*_, with amplitude *a*_*u*_*d*__ = 0.2 (A and C), *a*_*u*_*d*__ = −0.2 (B), is applied at time *t* = 1. (D) Input-output (*I*-*O*) relationships for the *U*_*NF*_ controller (solid magenta plot), its approximation by the saturation function sat(*m* ⋅ *e*) with *m* = *n*/(4*K*) (dashed blue plot), and the ideal SMC (i.e. the discontinuous nonlinearity sgn(*e*)—dashed-dotted green plot). When *n* = 2, then the saturation function sat(*e*/(2*K*)) is equal to the piecewise function *u*_*pw*_ that approximates *U*_*NF*_ (see Eqs (A38)–(A39) in S1 Appendix).

We now show how the ultrasensitive controller defined by Eq (17) implements an approximation of a well-known class of controllers, based on sliding mode control (SMC), a nonlinear technique for robust control system design [32–34]. Note first that as *K* goes to zero, Eq (17) assumes the following formula (see also Fig 6D):

$$\begin{array}{c} u\left(t\right)=k_{p}\text{sgn}\left(e\left(t\right)\right)\;. \end{array}$$ (18)

This kind of switching controller takes only two values, *k*_*p*_ and −*k*_*p*_, and has a discontinuity on the straight line *e* = 0. The equation of the line, *σ* = *e* = *r* − *y* = 0, is known in sliding mode control theory as the sliding manifold, where *σ* is the sliding variable. The typical dynamics in sliding mode control consist of a *reaching phase*, during which trajectories starting away from the sliding manifold *σ* = 0 move towards it and reach it in finite time, followed by a *sliding phase*, during which the dynamics will be confined to the manifold *σ* = 0. By setting opportunely the gain *k*_*p*_ (for details see S1 Appendix), the control signal *u*, defined by Eq (18), will therefore bring the error to zero in finite time and then maintain the condition *σ* = 0 for all future time.

Fig 6A shows the performance of the closed loop system with a sliding mode controller described by Eq (18) for different values of *k*_*p*_. We assume that the system output is initially equal to the desired constant output, *r* = *y*(0) = *y*_0_ = *v*_0_ = 1 (*L* = 0 in inequality (A33) in S1 Appendix). Then the control gain is only designed to compensate for the bounded disturbance *u*_*d*_, which is assumed to be a step disturbance applied at time *t* = 1 with amplitude *a*_*u*_*d*__ = 0.2. So setting *k*_*p*_ > *a*_*u*_*d*__ (see relation (A33) in S1 Appendix) (the parameter *a* = 1) is sufficient to reduce completely the effect of the disturbance *u*_*d*_ on the output *y* and to obtain the desired response (red dashed-dotted plot in Fig 6A). By contrast, if *k*_*p*_ < *a*_*u*_*d*__, the system is not able to attenuate the effects of the disturbance (blue plot in Fig 6A).

Fig 6B shows the performance when the output (*y*(0) = *y*_0_ = 0) is not initially equal to the desired value (*r* = 1) and a step disturbance is applied at time *t* = 1 with amplitude *a*_*u*_*d*__ = −0.2. Then the control gain of the sliding mode controller is designed not only to compensate for the disturbance, but also to force the system to move toward the sliding manifold (*σ* = *e* = 0) (in this case *L* > 1 in inequality (A33) in S1 Appendix). As shown in Fig 6B, if inequality (A33) in S1 Appendix is satisfied, then the system is able to move towards the sliding manifold *σ* = *e* = 0 and maintain it for all future time.

Fig 6A and 6B show that the output response exhibits a zigzag motion of small amplitude and high frequency in the sliding mode, a phenomenon known as *chattering*. For an ideal SMC, the switching frequency goes to infinity and the amplitude of the zigzag motion goes to zero—note, however, that such an infinitely fast switching frequency is not achievable in biological reality. In addition, theoretical issues like the existence and uniqueness of solutions and validity of the Lyapunov analysis (see S1 Appendix) have to be considered due to the discontinuous nonlinearity sgn(*e*) in the ideal SMC (see Eq (18)).

In engineering practice, therefore, these issues are usually avoided by using continuous/smooth approximations of the discontinuous SMC. Interestingly, the *U*_*NF*_ controller considered here is an example of such a smooth control function, which can be used to approximate the nonlinearity sgn(*e*). In this case, there is no ideal sliding mode in the closed-loop system of Fig 5A, since the sliding variable cannot be driven to zero in a finite time. However, for small values of *K*, the closed-loop response of the system with an *U*_*NF*_ controller is close to that achieved by an ideal SMC (Fig 6C). Moreover, Eq (17) can be approximated by the following saturation nonlinearity with high slope

$$\begin{array}{c} \text{sat}\left(m\cdot e\right)=\left\{\begin{array}{cc} m\cdot e\;, & |e|\leq 1/m,\\ \text{sgn}(m\cdot e)\;, & |e|>1/m,\; \end{array}\right. \end{array}$$ (19)

where *m* is the slope for the linear regime. Fig 6D shows the sigmoidal input-output relationship for the *U*_*NF*_ controller with *K* = 0.01, *n* = 2 and *k*_*p*_ = 1, together with its saturation function approximation and the discontinuous nonlinearity sgn(*e*). More generally, the *U*_*NF*_ controller can be approximated by a piecewise linear function [55] and, when *K* becomes small, the piecewise linear function is well-approximated by the corresponding saturation function (see Eqs (A37)–(A39) in S1 Appendix).

Fig 6C shows the output response of the closed-loop system for the *U*_*NF*_ controller with *n* = 2 and different values of *K*, together with the response obtained using its approximated saturation function (defined by Eq (A39) in S1 Appendix) for the same set of *K* values, and the response given by the ideal SMC. As shown in the figure, only the ideal SMC is able to completely eliminate the effect of the disturbance. However, the results for the *U*_*NF*_ and its approximated controller are very similar and the effect of the disturbance on the output becomes negligible by decreasing the *K* value of the *U*_*NF*_ (i.e increasing the slope *m* = 1/(2*K*) of the saturation function).

Indeed, as shown in [33], the saturation control function of Eq (19) will force the trajectory of the closed-loop system to reach in finite time the set |*e*| < 1/*m*, called the *boundary layer*, and remain inside it thereafter. Therefore, the ideal discontinuous SMC is replaced by a smooth/continuous controller, such that the error is not confined to the manifold *e* = 0, but lies inside the boundary layer |*e*| < 1/*m*. In the case of the *U*_*NF*_ controller, a good estimate of the boundary layer is given by

$$\begin{array}{c} |e|<(4K)/n\;. \end{array}$$ (20)

Since the boundary layer specifies the maximum value of the error signal, relation Eq (20) can be used as design guidelines to relate controller parameters (that need to be chosen by the designer) to closed-loop performance. In the limit *K* → 0 (*m* → ∞), the dynamics of the *U*_*NF*_ controller approach those of the ideal SMC. Thus, the *U*_*NF*_ controller of Eq (17) is an example of a *quasi sliding mode controller*, for which a large set of supporting theoretical results and computer-aided design tools exist in the engineering literature [34].

## Discussion

As Synthetic Biologists strive to design and build ever more complex systems, it is imperative to make progress in linking feedback control theory with the mechanistic realities underlying cellular information processing. Ultrasensitivity and negative feedback are ubiquitous features of biomolecular circuitry—when combined they offer the potential for achieving precise, fast and robust control over biomolecular dynamics—and there is increasing evidence that such ultrasensitive negative feedback is a core control strategy employed by natural biological control systems. Indeed, ultrasensitivity is observed to emerge via many different mechanisms including covalent modification, dimerization and branching architectures [4, 6, 8]. The yeast osmoregulation system implements the archetypical MAPK pathway, as well as a two-component signalling system, both of which control downstream gene expression, and have been shown theoretically and experimentally to embed ultrasensitive dynamics [4, 5, 28–31], while gene expression dynamics are usually implemented as a Hill function (e.g. see [27]). Moreover, the presence of ultrasensitivity has also been suggested for the Fps1 glycerol channels [25, 27]. Given such prevelance of ultrasensitivity in the osmosensing system, the findings presented here suggest that *U*_*NF*_ could be an appropriate paradigm for understanding cellular adaptive response dynamics. In the *E. coli* chemotaxis system, which also displays adaptive responses, ultrasensitivity is observed at the level of receptors and in the interaction of the signalling proteins with the motility apparatus [56, 57]. It is thus also possible that ultrasensitivity is implemented within the chemotaxis signalling pathways [8] and might have underpinned an evolutionary step in generating adaptive response dynamics [18, 58].

Moreover, we show that the *U*_*NF*_ controller can approximate a sliding mode controller, whose strong robustness and performance properties are well understood amongst control theorists [32–34]. It has so far not been appreciated that ultrasensitivity, when combined with negative feedback, can implement a quasi sliding mode controller in order to generate adaptive responses. Here, we explore this connection in detail and derive a direct relationship between the key property of the quasi sliding-mode controller (the boundary layer specifying the maximum values of the error signal), and the biologically inspired parameters of the *U*_*NF*_ controller (*n* and *K*) (see relation Eq (20)). Then, if the yeast osmoregulation system employs an *U*_*NF*_ controller, our study shows that adaptation precision would depend on the parameters *n* and *K*. Thus, we could experimentally evaluate the presence/absence of an *U*_*NF*_ controller by measuring changes in adaptation precision with regard to changes in parameters *n* and *K* describing the sensitivity and threshold of the ultrasensitive response. Biologically, the values of these parameters would depend on the kinetic rates and structure of the biochemical reactions implementing ultrasensitivity in the yeast osmoregulation system, as discussed above. In particular, it has been shown both theoretically and experimentally that the level of sensitivity (i.e. parameter *n*) and the level of signal threshold (i.e. parameter *K*) in MAPK signalling systems can be controlled by the concentration and kinetic properties of kinases and phosphatases [4], and the level of scaffolding proteins [59, 60]. Similarly, it is shown that two-component signalling cascades are able to implement ultrasensitivity, in a manner where both sensitivity and threshold level can be tuned by the concentration of the proteins involved [31, 44, 61]. Thus, experimentally altering the structure and protein concentrations of the MAPK and two-component signalling cascades in the yeast osmoregulation system would be expected to lead to alterations in the adaptation precision of the cell volume and Hog1 levels following an osmoshock if the system implements an *U*_*NF*_ controller.

In synthetic biology, where the aim is *de novo* engineering of system dynamics, then the *U*_*NF*_ controller provides a simple way for implementing adaptive response dynamics. The engineering of an *U*_*NF*_ controller can make use of several mechanisms for implementing ultrasensitivity, including those observed from dimerization of transcription factors [6], use of scaffolding proteins in MAPK systems [7], and branching in bacterial phosphorylation systems [8]. In the case of the ubiquitous phosphorylation-dephosphorylation cycles, several biochemical implementations have already been identified theoretically as implementing adaptive response dynamics [16, 18–20]. Therefore, by recognizing the important role of ultrasensitivity and negative feedback control in generating adaptive response dynamics in biological systems, and making connections between these biological realities and a branch of nonlinear control theory known as sliding mode control, we are able to generate analytical insights and quantitative design guidelines that provide a useful foundation for progressing the design and construction of robust synthetic feedback control systems.

## Supporting Information

S1 File A zipped folder containing the MATLAB code for generating the results presented in the main text and Supporting Information. (ZIP)

S1 Appendix Performance analysis of *P*_*NF*_, *I*_*NF*_, *FI*_*NF*_ and *U*_*NF*_ controllers using a generic closed-loop feedback system. (PDF)

S1 Fig Best fit to osmoshocks for the *P*_*NF*_-*P*_*NF*_ model. Best fit to the experimental dataset for the cell volume (A) and the Hog1 (B) responses to three step osmoshocks of different magnitude; the experimental data for 0.2, 0.4, and 0.6 M of NaCl are indicated by black circles, red diamonds, and blue squares, respectively. The corresponding coloured solid lines represent the optimised model responses.
(EPS)

S2 Fig Best fit to osmoshocks for the *P*_*NF*_-*I*_*NF*_ model. Best fit to the experimental dataset for the cell volume (A) and the Hog1 (B) responses to three step osmoshocks of different magnitude; the experimental data for 0.2, 0.4, and 0.6 M of NaCl are indicated by black circles, red diamonds, and blue squares, respectively. The corresponding coloured solid lines represent the optimised model responses.
(EPS)

S3 Fig Performance evaluation for the *P*_*NF*_-*I*_*NF*_ controller by varying the value of the optimized integral control gain, *k*_*HOG*_. (A) Volume and Hog1 responses to increasing the optimized value of *k*_*HOG*_. (B) Volume and Hog1 responses to decreasing the optimized value of *k*_*HOG*_. In each panel the upper plots show the volume responses; the lower plots show the Hog1 responses to step osmoshocks of 0.2 (first column), 0.4 (second column) and 0.6 M (third column) of NaCl. In each plot the red circles represent the experimental data.
(EPS)

S4 Fig Best fit to osmoshocks for the *P*_*NF*_-*FI*_*NF*_ model. (A-F) Best fit to the experimental dataset, the cell volume (A, C, E) and the Hog1 (B, D, F) responses to three step osmoshocks of different magnitude, with different values of the integration time window, *T*_*m*_ (*T*_*m*_ = 5 min (A, B); *T*_*m*_ = 10 min (C, D); *T*_*m*_ = 20 min (E, F)). The experimental data for 0.2, 0.4 and 0.6 M of NaCl are indicated by black circles, red diamonds, and blue squares, respectively. The correspondingly colored solid lines represent the optimized model responses.
(EPS)

S5 Fig Best fit to osmoshocks for the *U*_*NF*_-*I*_*NF*_ model. Best fit to the experimental dataset for the cell volume (A) and the Hog1 (B) responses to three step osmoshocks of different magnitude; the experimental data for 0.2, 0.4, and 0.6 M of NaCl are indicated by black circles, red diamonds, and blue squares, respectively. The corresponding coloured solid lines represent the optimised model responses.
(EPS)

S6 Fig Best fit to osmoshocks for the *U*_*NF*_-*FI*_*NF*_ model. (A-F) Best fit to the experimental dataset, the cell volume (A, C, E) and the Hog1 (B, D, F) responses to three step osmoshocks of different magnitude, with different values of the integration time window, *T*_*m*_ (*T*_*m*_ = 5 min (A, B); *T*_*m*_ = 10 min (C, D); *T*_*m*_ = 20 min (E, F)). The experimental data for 0.2, 0.4 and 0.6 M of NaCl are indicated by black circles, red diamonds, and blue squares, respectively. The correspondingly colored solid lines represent the optimized model responses.
(EPS)

S7 Fig Responses to different step osmoshocks by adding normally distributed noise to the controller outputs for the *U*_*NF*_-*U*_*NF*_ model. (A) Volume and (B) Hog1 responses to three step osmoshocks of different magnitude. Black plots: simulated responses to a step osmoshock of 0.2 M of NaCl (grey circles—experimental data); red plots: simulated responses to a step of 0.4 M of NaCl (grey diamonds—experimental data); blue plots: simulated responses to a step of 0.6 M of NaCl (grey squares—experimental data). 1000 independent simulations are performed.
(EPS)

S8 Fig Performance of the generic closed-loop feedback system for *P*_*NF*_, *I*_*NF*_ and *FI*_*NF*_ controllers. Response dynamics obtained using: (A) *P*_*NF*_ for different values of the gain *k*_*p*_; (B) *I*_*NF*_ for different values of the gain *k*_*i*_; (C-E) *FI*_*NF*_ with the time window *T*_*m*_ = *τ* and different values of the gain *k*_*i*_ (C); *T*_*m*_ = 5*τ* and different values of *k*_*i*_ (D); *k*_*i*_ = 1 and different values of *T*_*m*_ (E). The system output is initially equal to the desired constant reference value *y*(0) = *y*_0_ = *r* = 1. A step disturbance, *u*_*d*_, with amplitude *a*_*u*_*d*__ = 0.2 is applied at time *t* = 1.
(EPS)

S1 Table Model parameters for the osmoregulation system. (PDF)

S2 Table Optimization of the model parameters for the different control schemes against multiple experimental datasets. (PDF)
