# Supplementary material for: Ultrasensitive Negative Feedback Control: A Natural Approach for the Design of Synthetic Controllers
Source: PLoS One. 2016 Aug 18;11(8):e0161605. doi: 10.1371/journal.pone.0161605 (PMC5004582; doi:10.1371/journal.pone.0161605)
Supplement: S1 Appendix — (PDF) [file pone.0161605.s002.pdf]

S1 Appendix
Ultrasensitive Negative Feedback Control: A Natural Approach for the Design of Synthetic Controllers

Francesco Montefusco[*], Ozgur E. Akman[†], Orkun S. Soyer[‡]
and Declan G. Bates[§]

List of contents

1. Generic control model
   1.1. Proportional control (P_{NF})
   1.2. Integral control with infinite (I_{NF}) vs. finite integration period (FI_{NF})
   1.3. Ultrasensitive control (U_{NF}) as a quasi sliding mode control

- Supporting References

[*]Department of Information Engineering, University of Padova, Italy
[†]College of Engineering, Mathematics and Physical Sciences, University of Exeter, United Kingdom
[‡]School of Life Sciences, University of Warwick, United Kingdom
[§]School of Engineering, University of Warwick, United Kingdom

1 Generic control model

Let us assume the closed-loop feedback control system of Fig. 5A of the main text, where the controller modifies the state of the process to obtain the desired output response. The input of the controller is the error signal, e, defined as the difference between the desired output, r (called the reference signal), and the actual output of the system, y. Based on the error signal, the controller manipulates the input to the process, u, to reduce the effect of the disturbance, u_d, on the output y and therefore obtain the desired response.

To simplify the analysis, the process is a first order linear system described with the following differential equation model

$$\dot{v}(t) = -a\left(v(t) - v_0\right) + bu(t)\,, \tag{A1}$$

where $v(t) \in \mathbb{R}$ is the output of the process in open loop without assuming any disturbance, a and b are positive scalars, $u(t) \in \mathbb{R}$ is the input and v_0 is the equilibrium point for the process when the input is zero. The solution of Eq. (A1) is given by $v(t) = b(1 - e^{-at})/a$ (assuming $v(0) = v_0 = 0$). Therefore the important measures that characterise the response of the open loop system (without considering the feedback action of the controller C) are the steady state value, $G = b/a$, or static gain, and the time constant $\tau = 1/a$, that corresponds to the time taken for the output to reach 63% of its final value ($v(1/a) = b(1 - e^{-1})/a = 0.63b/a = 0.63G$). In the following we consider the case for $v_0 = 0$. The results are the same for $v_0 \neq 0$. Indeed, via the change of variable $z = v - v_0$, the system $\dot{z}(t) = -az(t) + bu(t)$ is equivalent to system (A1).

Now, the output of the closed loop system is given by

$$y(t) = v(t) + u_d(t)\,, \tag{A2}$$

where $u_d(t)$, the additive disturbance on the output of the process, is a step signal at time t_d ($u_d(t) = 0$ for $t < t_d$ and $u_d(t) = a_{u_d}$ for $t \geq t_d$, with a_{u_d} the disturbance amplitude).

1.1 Proportional control (P_{NF})

Let us assume that the controller C produces an output value, u, proportional to the current value of the error e

$$u(t) = k_p e(t) = k_p(r(t) - y(t))\,,$$

where k_p is a constant called the gain of the proportional controller.

The dynamics of the closed loop system (taking into account Eqs. (A1) and (A2)) are then described

by the following differential equation:

$$\begin{aligned}\dot{y}(t) = \dot{v}(t) + \dot{u}_d(t) =& - av(t) + bk_p(r(t) - y(t)) + \dot{u}_d(t)\\ =& - a(y(t) - u_d(t)) + bk_p(r(t) - y(t)) + \dot{u}_d(t)\,. \end{aligned} \tag{A3}$$

To solve this differential equation the Laplace operator is used, since it allows differential equations to be transformed into algebraic equations that are much easier to solve (see (1); in particular note that the derivative operator with respect to time corresponds to a multiplication by s in the s-domain, where s is the complex Laplace variable). Taking the Laplace transform of Eq. (A3) and assuming a zero initial condition gives

$$\begin{aligned} sY(s) &= -a(Y(s) - U_d(s)) + bk_p(R(s) - Y(s)) + sU_d(s)\,,\\ \Longrightarrow\ (s + a + bk_p)Y(s) &= bk_pR(s) + (s + a)U_d(s)\,, \end{aligned}$$

where $R(s)$, $Y(s)$ and $U_d(s)$ are the Laplace transforms of the reference, output and disturbance signals, respectively.

By applying the superposition principle (for a linear system the output response to two or more inputs is the sum of the responses which would have been caused by each input individually: see (1)), the transfer function W_{yr}, defined as the ratio of the Laplace transform of the output y and the desired output r with $u_d(t) = 0$, is given as

$$W_{yr}(s) = \frac{Y(s)}{R(s)} = \frac{bk_p}{s + a + bk_p}\,, \tag{A4}$$

and the transfer function W_{yu_d}, defined as the ratio of the Laplace transform of the output y and the disturbance u_d with $r(t) = 0$, is given as

$$W_{yu_d}(s) = \frac{Y(s)}{U_d(s)} = \frac{s + a}{s + a + bk_p}\,. \tag{A5}$$

Assuming that the desired signal is a constant, $r(t) = r$, and the disturbance is a step signal at time t_d ($u_d(t) = 0$ for $t < t_d$ and $u_d(t) = a_{u_d}$ for $t \geq t_d$, where a_{u_d} is the disturbance amplitude) the corresponding Laplace transforms are $R(s) = \frac{r}{s}$ and $U_d(s) = \frac{a_{u_d}e^{-t_d s}}{s}$. Then the Laplace transform of y, obtained by summing Eqs. (A4) and (A5), is given by

$$Y(s) = W_{yr}(s)R(s) + W_{yu_d}(s)U_d(s) = \frac{bk_p}{s + a + bk_p} \cdot \frac{r}{s} + \frac{s + a}{s + a + bk_p} \cdot \frac{a_{u_d}e^{-t_d s}}{s}\,. \tag{A6}$$

By applying the final-value theorem for Laplace transforms (2),

$$y_{ss} = \lim_{t\to\infty} y(t) = \lim_{s\to 0} sY(s)\,, \tag{A7}$$

where y_{ss} is the steady state value of the output, the input-output steady states are related via

$$\begin{aligned} y_{ss} =& \lim_{s\to 0}\left(sW_{yr}(s)\frac{r}{s} + sW_{yu_d}(s)\frac{a_{u_d}e^{-t_d s}}{s}\right) \\ =& W_{yr}(0)r + W_{yu_d}(0)a_{u_d} = \frac{bk_p}{a+bk_p}r + \frac{a}{a+bk_p}a_{u_d} \\ =& \frac{Gk_p}{1+Gk_p}r + \frac{1}{1+Gk_p}a_{u_d}\,. \end{aligned} \tag{A8}$$

Note that the last term of Eq. (A8) is obtained by dividing numerator and denominator by a. Therefore, if $Gk_p >> 1$, the output y is able to track the desired reference signal r (i.e. $y_{ss} \sim r$) and the controller is able to attenuate the effects of the disturbance u_d on y. In terms of the error, Eq. (A8) can be rewritten as

$$e_{ss} = r - y_{ss} = \frac{1}{1+Gk_p}r - \frac{1}{1+Gk_p}a_{u_d}\,. \tag{A9}$$

The dynamics of the closed-loop system are determined by the pole of the transfer functions W_{yr} and W_{yu_d} (the value p of s that nullifies the common denominator of Eqs. (A4) and (A5), i.e. $s = p = -a - bk_p$). The time constant τ_c of the dynamics is given by

$$\tau_c = -\frac{1}{p} = \frac{1}{a+bk_p} = \frac{\tau}{1+Gk_p}\,, \tag{A10}$$

where $\tau = 1/a$ is the time constant of the open loop system. Thus, the output response becomes faster for high values of k_p than the response of the open loop system without the controller.

Panel A in S8 Fig shows the performance of the closed loop system with different values of k_p. As expected from Eqs. (A8)–(A10), increasing the gain value k_p allows the system to attenuate the effects of the disturbance and achieve a faster response.

1.2 Integral control with infinite (I_{NF}) vs. finite integration period (FI_{NF})

In this section we consider the closed loop system of Fig. 5A of the main text with a constant reference signal r, a step disturbance u_d of amplitude a_{u_d} and a controller implementing an integrator with an infinite or finite integration period.

It is well known that integral control with infinite integration period (I_{NF}) can be used to completely attenuate the effects of the disturbance u_d on the desired output y of the closed loop system

of Fig. 5A of the main text: in fact, y is able to perfectly track any step reference signal, r, for any step disturbance, u_d (1). In this case the system is able to achieve an adaptive response. In general, in order to achieve an adaptive response to a reference signal corresponding to the n-th integral of a step function, where n is a positive integer, the feedback loop has to contain at least $n + 1$ integrators connected in series (2). Adaptive responses to step signals, where $n = 0$, require at least one integrator.

For such a system, the controller C produces an action which takes into account completely the history of the process with an output value, u, proportional to the integral of the error

$$u(t) = k_i \int_0^t e(\tau)d\tau = k_i \int_0^t (r(\tau) - y(\tau))d\tau,$$

where k_i is the gain of the integrator. The response of the closed loop system is obtained by solving the following integro-differential equation:

$$\dot{y}(t) = -a(y(t) - u_d(t)) + bk_i \int_0^t (r(\tau) - y(\tau))d\tau + \dot{u}_d(t). \tag{A11}$$

Taking the Laplace transform of Eq. (A11) and noting that the integral operator with respect to time corresponds to division by s in the s-domain (1) gives

$$sY(s) = -a(Y(s) - U_d(s)) + bk_i \left(\frac{R(s)}{s} - \frac{Y(s)}{s} \right) + sU_d(s)\,,$$

where $R(s)$, $Y(s)$, $U_d(s)$ are the Laplace transforms of the reference, output, input and disturbance signals, respectively. It follows that the transfer functions, $W_{yr}(s)$ and $W_{yu_d}(s)$, are given by

$$W_{yr}(s) = \frac{Y(s)}{R(s)} = \frac{bk_i}{s^2 + as + bk_i}\,, \tag{A12}$$

$$W_{yu_d}(s) = \frac{Y(s)}{U_d(s)} = \frac{s(s + a)}{s^2 + as + bk_i}\,. \tag{A13}$$

By applying the final-value theorem, the input-output and error steady-states are related via

$$y_{ss} = W_{yr}(0)r + W_{yu_d}(0)a_{u_d} = \frac{bk_i}{bk_i} r + 0 = r\,, \tag{A14}$$

$$e_{ss} = r - y_{ss} = 0\,. \tag{A15}$$

Therefore the steady state output y_{ss} is equal to r for any value of the gain k_i. In other words, the integral feedback control allows the system to perfectly return to its steady state value (i.e. achieve

perfect adaptation) following any value of perturbation.

The gain k_i determines the time dynamics of the system, i.e. the time taken for the system to reach steady state following a perturbation. In particular, the poles of the two transfer functions $W_{yr}(s)$ and $W_{yu_d}(s)$ (the values of s that nullify the denominator of Eqs. (A12) and (A13)) determine the evolution of the system. The denominator of the two functions, $s^2 + as + bk_i$, can be rewritten in the standard form

$$s^2 + 2\zeta\omega_n s + \omega_n^2 \,, \tag{A16}$$

where $\omega_n = \sqrt{\frac{b}{a}ak_i} = \sqrt{Gk_i/\tau}$ is called the natural frequency of the system and $\zeta = \frac{a}{2\sqrt{Gk_i/\tau}} = \frac{1}{2\sqrt{Gk_i\tau}}$ is called the damping factor (1). The natural frequency gives information on the speed of the system response; the damping factor combines the initial speed of the response and its accuracy defined in terms of overshoot and settling time. For $\zeta < 1$ the system is underdamped (presence of overshoot in the response) and as ζ decreases the system exhibits a faster response, but more oscillatory behaviour. For $\zeta > 1$ the system is overdamped (i.e. no overshoot phenomena) and as ζ increases the response becomes slower. For $\zeta = 1$ (*critical damping*) the system responds with the maximum speed without overshoot (1).

Panel B in S8 Fig shows the performance of I_{NF} with different values of k_i. The system perfectly adapts for any value of k_i, which determines the response dynamics: for small values of k_i the response is slow, whereas for higher values the system achieves faster responses but with more oscillatory behaviour.

So far we have assumed a controller implementing I_{NF}, an ideal integrator that is able to store the full history of the process. We now consider a form of integral feedback that implements a finite window for error integration (FI_{NF}). For FI_{NF}, the controller output signal u is given by

$$u(t) = k_i \int_{t-T_m}^{t} e(\tau)d\tau = k_i \int_{t-T_m}^{t} (r(\tau) - y(\tau))d\tau \,,$$

where T_m is the time window of the integral. The following integro-differential equation then describes the closed loop system:

$$\dot{y}(t) = -a(y(t) - u_d(t)) + bk_i \int_{t-T_m}^{t} (r(\tau) - y(\tau))d\tau + \dot{u}_d(t) \,. \tag{A17}$$

Taking the Laplace transform of Eq. (A17) and remembering that time-shifting corresponds to expo-

nential multiplication in the Laplace domain gives

$$sY(s) = -a(Y(s) - U_d(s)) + bk_i \left(\frac{R(s)}{s} \left(1 - e^{-sT_m}\right) - \frac{Y(s)}{s} \left(1 - e^{-sT_m}\right) \right) + sU_d(s) \, .$$

It follows that the transfer functions, $W_{yr}(s)$ and $W_{yu_d}(s)$, are given by

$$W_{yr}(s) = \frac{Y(s)}{R(s)} = \frac{bk_i(1 - e^{-sT_m})}{s^2 + as + bk_i(1 - e^{-sT_m})} \, , \tag{A18}$$

$$W_{yu_d}(s) = \frac{Y(s)}{U_d(s)} = \frac{s(s + a)}{s^2 + as + bk_i(1 - e^{-sT_m})} \, . \tag{A19}$$

By applying the final-value theorem, the input-output and error steady-states are related via

$$\begin{aligned} y_{ss} =& \lim_{s \to 0} \left(sW_{yr}(s)\frac{r}{s} + sW_{yu_d}(s)\frac{a_{u_d} e^{-t_d s}}{s} \right) \\ =& \frac{bk_iT_m}{a + bk_iT_m} r + \frac{a}{a + bk_iT_m} a_{u_d} \\ =& \frac{Gk_iT_m}{1 + Gk_iT_m} r + \frac{1}{1 + Gk_iT_m} a_{u_d} \, , \end{aligned} \tag{A20}$$

$$e_{ss} = r - y_{ss} = \frac{1}{1 + Gk_iT_m} r - \frac{1}{1 + Gk_iT_m} a_{u_d} \, . \tag{A21}$$

(Note that as $s \to 0$, the term e^{-sT_m} can be approximated by the first two terms of its Taylor series ($e^{-sT_m} \sim 1 - sT_m$)).

Panel C in S8 Fig shows the performance of FI_{NF} with a time window $T_m = \tau$ for different values of k_i. The results are similar to those obtained from the proportional controller: increasing the gain k_i reduces the effect of the disturbance on the output, but the steady state never returns to the exact value prior to the perturbation. The controller acts similarly to an ideal integrator when its time window is sufficiently large compared to the dynamics of the process to be controlled: panel D in S8 Fig shows the performance of FI_{NF} with $T_m = 5\tau$ for different values of k_i. The control system tries to cancel the effect of the disturbance but the response is characterised by a more pronounced oscillatory behaviour which does not damp down, like the ideal integrator, due to the finite memory T_m. Finally, panel E in S8 Fig shows the performance of FI_{NF} as the window time T_m is varied for a fixed gain k_i equal to 1. As shown previously, for small values of T_m the results are similar to those of the proportional control model, whereas for large values of T_m the system reduces the effect of the disturbance but generates an oscillatory response, which does not die out like in the case of I_{NF}.

1.3 Ultrasensitive control (U_{NF}) as a quasi sliding mode control

Ultrasensitivity describes a particular form of sensitivity in biological systems, where the system does not respond to incoming signals outside of a certain regime, but responds in a highly sensitive manner within this regime. Ultrasensitivity is characterised by a sigmoidal input-output relationship and is shown to be a ubiquitous feature in several biological systems. Ultrasensitivity can be biochemically implemented through a variety of mechanisms such as phosphorylation cycles and cooperative binding (see (3, 4)) and can be described by a Hill-type function. It has been shown previously that ultrasensitivity, when implemented in a negative feedback loop, can lead to adaptive response dynamics (5).

Biochemical reactions that can implement ultrasensitivity in biological systems can take part in particular signalling and response pathways. To represent these systems using the control theory framework developed above, we imagine an ultrasensitive controller (U_{NF}). In this controller, the error is processed through an ultrasensitive (sigmoidal dose-response) dynamics, before being fed into the process. Thus, the input from the controller back to the process can be represented by

$$u(t) = \mathrm{sgn}(e(t))k_p \cdot \frac{|e(t)|^n}{|e(t)|^n + K^n} \,. \tag{A22}$$

Note that we use the sign and absolute value of the error to allow the controller to work in a symmetrical way for positive and negative values of the error.

The dynamics of the closed loop system are therefore described by the following differential equation:

$$\dot{y}(t) = -a(y(t) - u_d(t)) + \mathrm{sgn}(e(t))bk_p \cdot \frac{|e(t)|^n}{|e(t)|^n + K^n} + \dot{u}_d(t)) \,. \tag{A23}$$

Rewriting Eq. (A23) in terms of e and assuming a constant reference signal, $r(t) = r$ (note that $\dot{e}(t) = -\dot{y}(t)$), gives

$$\dot{e}(t) = a(r - e(t) - u_d(t)) - \mathrm{sgn}(e(t))bk_p \cdot \frac{|e(t)|^n}{|e(t)|^n + K^n} - \dot{u}_d(t)) \,. \tag{A24}$$

The steady state error, e_{ss}, is the solution of the equation $\dot{e}(t) = 0$ which implies

$$a(r - e_{ss} - a_{u_d}) = \mathrm{sgn}(e_{ss})bk_p \cdot \frac{|e_{ss}|^n}{|e_{ss}|^n + K^n} \,. \tag{A25}$$

The solutions of Eq. (A25) can be visualised by plotting both sides of the equation, as shown in Fig. 5B of the main text: the intersections of the straight line $a(r - e - a_{u_d})$ with $\mathrm{sgn}(e)bk_p \cdot \frac{|e|^n}{|e|^n + K^n}$ correspond to the steady states of the system. We show that U_{NF} can achieve a steady-state error value very close to zero for any value of n, given a small value of K and without increasing the gain k_p (for more

details see the *Results* section of the main text).

From the control point of view, the ultrasensitive controller defined by Eq. (A22) is an approximation of a well-known class of controllers, based on sliding mode control (SMC), a nonlinear technique for robust control (see (6–8)). Indeed if K goes to zero, Eq. (A22) assumes the following formula (see also Fig. 6D of the main text):

$$u(t) = k_p \mathrm{sgn}(e(t)) \,. \tag{A26}$$

As explained in the main text, this kind of controller is a simple switching control that takes only two values, k_p and $-k_p$, and has a discontinuity on the straight line $e = 0$. The equation of the line, $\sigma \stackrel{\text{def}}{=\!=} e = r - y = 0$, may be interpreted as the sliding mode equation, called the sliding manifold, where σ is the sliding variable. The typical dynamics in sliding mode control consist of a *reaching phase* during which trajectories starting off the sliding manifold $\sigma = 0$ move towards it and reach it in finite time, followed by a *sliding phase* during which the dynamics will be confined to the manifold $\sigma = 0$. The control signal u, defined by Eq. (A26), is therefore designed to bring the error to zero in finite time and then maintain the condition $\sigma = 0$ for all future time. This task can be achieved by applying Lyapunov stability (1, 7, 8) to the σ-dynamics, defined as

$$\dot{\sigma}(t) = \dot{e}(t) = -\dot{y}(t) = a(y(t) - u_d(t)) - bu(t) - \dot{u}_d(t) = a(r - e(t) - u_d(t)) - bk_p \mathrm{sgn}(e(t)) - \dot{u}_d(t) \,. \tag{A27}$$

For the σ-dynamics, a candidate Lyapunov function is

$$V = \frac{1}{2}\sigma^2 \,. \tag{A28}$$

In order for $\sigma = 0$ to be asymptotically stable, the following conditions need to be satisfied:

$$V(\sigma) > 0 \,, \tag{A29}$$

$$\dot{V}(\sigma) < 0 \quad \text{for} \quad \sigma \neq 0 \,. \tag{A30}$$

The condition (A29) is satisfied by Eq. (A28). The derivative of Eq. (A28) is computed as

$$\dot{V} = \sigma\dot{\sigma} = e\dot{e} = e(-\dot{y}) = e\left(a(y - u_d) - bu - \dot{u}_d\right) = e\,\left(a(r - e - u_d) - bk_p \mathrm{sgn}(e) - \dot{u}_d\right) . \tag{A31}$$

To attain the sliding manifold, $\dot{V} < 0$, σ and $\dot{\sigma}$ must always have opposite signs. Assuming that u_d

and $e(0)$ are bounded as below

$$|u_d(t)| < M \quad \text{and} \quad |e(0)| = |r - y(0)| < L\,, \tag{A32}$$

the product $\sigma\dot{\sigma}$ will be negative if the following inequality is satisfied:

$$k_p\, b \geq a\, M + a\, L \implies k_p \geq \frac{a}{b}M + \frac{a}{b}L\,. \tag{A33}$$

Therefore, the control gain k_p of the SMC controller is computed taking into account the relation (A33); the first term, $\frac{a}{b}M$, is designed to compensate for the bounded disturbance u_d, while the second term $\frac{a}{b}L$ is responsible for determining the reaching phase, when the trajectory is forced towards the sliding manifold.

Note that, if the controller can also make an action proportional to the output, then the inequality (A33) for the control gain k_p is only determined by the disturbance u_d, i.e. $k_p \geq \frac{a}{b}M$. In particular, if the input at the process given by the controller is

$$\bar{u}(t) = k_p \text{sgn}(e(t)) + \frac{a}{b}y(t)\,, \tag{A34}$$

then Eq. (A31) becomes

$$\dot{V} = e\left(a(y - u_d) - b\bar{u} - \dot{u}_d\right) = e\left(ay - au_d - bk_p\text{sgn}(e) - ay - \dot{u}_d\right) = e\left(-au_d - bk_p\text{sgn}(e) - \dot{u}_d\right). \tag{A35}$$

Inequality (A33) thus becomes $k_p \geq \frac{a}{b}M$; the term $u_{eq} = ay/b$ is called *equivalent control* and is chosen to cancel the known term, ay, on the right-hand side of Eq. (A27). In the absence of the disturbance, taking $u = u_{eq}$ leads to $\dot{e} = 0$, allowing the sliding manifold $\sigma = e = 0$ to be maintained for all future time.

As shown in Fig 6 of the main text, the output response of the closed loop system with a SMC exhibits a zigzag motion of small amplitude and high frequency in the sliding mode (see the plot in the box of Fig. 6A). SMC is a high frequency switching control with a switching frequency inversely proportional to the time increment used in the simulation. Increasing the accuracy of the solution computed by the ODE solver reduces the zigzag motion, but due to the discrete-time nature of the computer simulation the output response continues to exhibit this effect, which is called *chattering*. For an ideal SMC, the switching frequency goes to infinity and the amplitude of the zigzag motion goes to zero.

In addition to the practical issue of chattering, theoretical issues like the existence and uniqueness of solutions and validity of the Lyapunov analysis have to be considered due to the discontinuous nonlinearity sgn(e) in the ideal SMC (see Eq. (A26)). These issues can be avoid by using continuous/smooth approximations of the discontinuous SMC and the U_{NF} controller is an example of a smooth control function, used to approximate the nonlinearity sgn(e). The designed U_{NF} control is technically not a sliding mode control and there is no ideal sliding mode in the closed-loop system (A23), since the sliding variable cannot be driven to zero in a finite time. However, for small values of K, the results of U_{NF} are close to those achieved by an ideal SMC (see Fig S8C). As shown in the main text, Eq. (A22) can be approximated by the following saturation nonlinearity with high slope

$$\mathrm{sat}(m\cdot e) = \begin{cases} m\cdot e\,, & |e|\le 1/m,\\ \mathrm{sgn}(m\cdot e)\,, & |e|>1/m, \end{cases} \tag{A36}$$

where m is the slope for the linear regime.

In wider terms, the U_{NF} controller can be approximated by a piecewise linear function (9) and, when K becomes small, the piecewise linear function is well-approximated by the corresponding saturation function. In particular, for $n<2$, the following approximation is valid

$$u = \mathrm{sgn}(e)\cdot\frac{|e|^n}{|e|^n+K^n} \approx u_{pw} = \begin{cases} n/(2K)\,e\,, & |e|\le K(2-n)/n,\\ n/(4K)\,e\,-1/4(2-n), & -K(2+n)/n < e \le -K(2-n)/n,\\ n/(4K)\,e\,+1/4(2-n), & K(2-n)/n < e \le K(2+n)/n,\\ \mathrm{sgn}(e)\,, & |e| > K(2-n)/n, \end{cases} \tag{A37}$$

while for $n\ge 2$

$$u = \mathrm{sgn}(e)\cdot\frac{|e|^n}{|e|^n+K^n} \approx u_{pw} = \begin{cases} 0\,, & |e|\le K(n-2)/n,\\ n/(4K)\,e\,-1/4(2-n), & -K(n+2)/n < e \le -K(n-2)/n,\\ n/(4K)\,e\,+1/4(2-n), & K(n-2)/n < e \le K(n+2)/n,\\ \mathrm{sgn}(e)\,, & |e| > K(n+2)/n. \end{cases} \tag{A38}$$

For $n=2$, the piecewise approximation u_{pw} (right-hand side of Eq. (A38)) is equal to the saturation function with $m=n/(4K)=1/(2K)$. In this case, the U_{NF} controller is thus well-approximated by

its saturation function:

$$u = \text{sgn}(e) \cdot \frac{|e|^2}{|e|^2 + K^2} \approx u_{pw} = \text{sat}(e/(2K)) = \begin{cases} e/(2K)\,, & |e| \le 2K, \\ \text{sgn}(e/(2K))\,, & |e| > 2K. \end{cases} \tag{A39}$$

As shown in Fig 6C of the main text, the U_{NF} controller and its approximated saturation function are very similar and the effect of the disturbance on the output becomes negligible by decreasing the K value of the U_{NF} (i.e increasing the slope $m = 1/(2K)$ of the saturation function for $n = 2$) and the error lies inside the boundary layer $|e| < 1/m$. Therefore, the ideal discontinuous SMC can be approximated by a smooth/continuous controller, such that the error is not confined to the manifold $e = 0$, but lies inside the boundary layer (7), that for the case of the U_{NF} controller, as reported in the main text, is given by

$$|e| < (4K)/n\,. \tag{A40}$$

In the limit $K \to 0$ ($m \to \infty$), the U_{NF} controller (the saturation function $\text{sat}(ne/(4K))$) approaches the signum nonlinearity $\text{sgn}(e)$ of the ideal SMC. This, finally, allows us to say that the U_{NF} controller of Eq. (A22) is an example of *quasi sliding mode control* (8).

Supporting References

1. Cosentino, C., and D. G. Bates, 2011. Feedback Control in Systems Biology. CRC Press (Taylor & Francis).

2. Skogestad, S., and I. Postlethwaite, 1996. Multivariable Feedback Control: Analysis and Design. Wiley.

3. Goldbeter, A., and D. E. Koshland, 1981. An amplified sensitivity arising from covalent modification in biological systems. *PNAS* 78:6840–6844.

4. Buchler, N. E., and F. R. Cross, 2009. Protein Sequestration Generates a Flexible Ultrasensitive Response in a Genetic Network. *Mol Syst Biol* 5:272.

5. Behar, M., N. Hao, H. G. Dohlman, and T. C. Elston, 2007. Mathematical and computational analysis of adaptation via feedback inhibition in signal transduction pathways. *Biophys J* 93:806–821. http://dx.doi.org/10.1529/biophysj.107.107516.

6. Utkin, V., 1992. Sliding Modes in Control and Optimization. Springer-Verlag, 1992.

7. Khalil, H., 2002. Nonlinear Systems. Prentice-Hall.

8. Shtessel, Y., C. Edwards, L. Fridman, and A. Levant, 2013. Sliding Mode Control and Observation. Springer.

9. Shin, Y.-J., and L. Bleris, 2010. Linear control theory for gene network modeling. *PLoS One* 5. http://dx.doi.org/10.1371/journal.pone.0012785.
