# Supplementary material for: Ultrasensitive Negative Feedback Control: A Natural Approach for the Design of Synthetic Controllers
Source: PLoS One. 2016 Aug 18;11(8):e0161605. doi: 10.1371/journal.pone.0161605 (PMC5004582; doi:10.1371/journal.pone.0161605)
Supplement: S1 Table — (PDF) [file pone.0161605.s011.pdf]

Model parameters: all volumes are scaled such that the initial volume of the cell is 1. Both Gly and Gly_e represent numbers of molecules (mol scaled by $V(0)$).

	Control Parameters	Bounds	References
k_{Fps1}	Glycerol permeability coefficient in a completely open Fps1 channel	[0.1 1]	[21, 25, 27, 38]
a_{HOG}, b_{HOG}	Hog1 system parameters	[0 1] Osm^{-1} min $^{-1}$	-
k_{HOG}	Hog1 control gain	[0 10]	-
n_{HOG}, n_{Fps1}	Hill exponents	[1 3]	[5]

	Biophysical Parameters	Bounds	References
$V^{P_t=0}$	V when $P_t = 0$	[0.5 0.99]	[22 and references therein, 26]
k_{p1}	Water permeability coefficient	[0.1 160] Osm^{-1} min $^{-1}$	[22 and references therein]

	Fixed Parameters	Bounds	Nominal Values	References
T_m	Time memory	[0 ∞]	5, 10 and 20 min	-
$k_{e_{Fps1}}$	Exponential constant	-	10	-
$k_{e_{HOG}}$	Exponential constant	-	10	-
$Gly(0)$	Initial Gly	[1.1 5]$\times 10^{-4}$	2×10^{-4}	[22 and references therein]
$P_i(0)$	Initial P_i	[0.6 0.7]	0.636 Osm	[22 and references therein]
$P_e(0)$	Initial P_e	[0.24 0.25]	0.24 Osm	[22 and references therein]
V_e	External volume	[0.5 5]$\times 10^3$	4.79×10^3	[22 and references therein]
V_b	Non-osmotic volume	[0.31 0.46]	0.368	[22 and references therein]

	Dependent parameters	Values
$V(0)$	Initial V - relative volume	1
$Gly_e(0)$	Initial Gly_e	$(V_e Gly(0))/(V(0) - V_b)$
$P_t(0)$	Initial P_t	$P_i(0) - P_e(0)$
s	No. of osmolytes other than Gly	$P_i(0)(V(0) - V_b) - Gly(0)$
