# Supplementary material for: Ultrasensitive Negative Feedback Control: A Natural Approach for the Design of Synthetic Controllers
Source: PLoS One. 2016 Aug 18;11(8):e0161605. doi: 10.1371/journal.pone.0161605 (PMC5004582; doi:10.1371/journal.pone.0161605)
Supplement: S2 Table — (PDF) [file pone.0161605.s012.pdf]

Optimized model parameters for $\mathbf{P_{NF}}$-$\mathbf{P_{NF}}$, $\mathbf{P_{NF}}$-$\mathbf{I_{NF}}$, $\mathbf{P_{NF}}$-$\mathbf{FI_{NF}}$, $\mathbf{U_{NF}}$-$\mathbf{I_{NF}}$, $\mathbf{U_{NF}}$-$\mathbf{FI_{NF}}$, $\mathbf{U_{NF}}$-$\mathbf{U_{NF}}$ and the corresponding scores obtained by fitting to multiple experimental datasets (volume and Hog1 responses to step shocks of 0.2, 0.4 and 0.6 M of NaCl). See S1 Table for the corresponding units.

Control scheme	Optimized values							
	k_{Fps1}	k_{HOG}	n_{Fps1}	n_{HOG}	b_{HOG}	a_{HOG}	$V^{P_t=0}$	k_{p1}
$\mathbf{P_{NF}}$-$\mathbf{P_{NF}}$	0.1	0.1561	1 (fixed)	1 (fixed)	0.4187	0.7317	0.8957	0.5512
$\mathbf{P_{NF}}$-$\mathbf{I_{NF}}$	0.1	0.0101	1 (fixed)	-	0.6068	0.5545	0.8213	142.2286
$\mathbf{P_{NF}}$-$\mathbf{FI_{NF}}$ ($T_m = 5$)	0.1	0.0477	1 (fixed)	-	0.2976	0.4135	0.8759	131.2170
$\mathbf{P_{NF}}$-$\mathbf{FI_{NF}}$ ($T_m = 10$)	0.1	0.0300	1 (fixed)	-	0.2401	0.2786	0.8610	105.2129
$\mathbf{P_{NF}}$-$\mathbf{FI_{NF}}$ ($T_m = 20$)	0.1	0.0148	1 (fixed)	-	0.3441	0.4319	0.8676	120.3590
$\mathbf{U_{NF}}$-$\mathbf{I_{NF}}$	0.2385	0.0045	1.7399	-	0.9423	0.9939	0.8276	157.7176
$\mathbf{U_{NF}}$-$\mathbf{FI_{NF}}$ ($T_m = 5$)	0.2657	0.0139	2.9876	-	0.7059	0.6967	0.8740	140.0372
$\mathbf{U_{NF}}$-$\mathbf{FI_{NF}}$ ($T_m = 10$)	0.1175	0.0073	2.9599	-	0.8695	0.8979	0.8577	100.9709
$\mathbf{U_{NF}}$-$\mathbf{FI_{NF}}$ ($T_m = 20$)	0.6736	0.0047	2.4606	-	0.8452	0.8905	0.8320	123.1382
$\mathbf{U_{NF}}$-$\mathbf{U_{NF}}$	0.1082	0.0172	2.9888	2.1847	0.7895	0.6613	0.8251	105.3189

Control scheme	Scores			
	J value defined by Eq (16) of the main text	AIC	BIC	FPE
$\mathbf{P_{NF}}$-$\mathbf{P_{NF}}$	3.8329	-1168	-1147	0.0145
$\mathbf{P_{NF}}$-$\mathbf{I_{NF}}$	0.9653	-1549	-1523	0.0037
$\mathbf{P_{NF}}$-$\mathbf{FI_{NF}}$ ($T_m = 5$)	3.5591	-1189	-1167	0.0135
$\mathbf{P_{NF}}$-$\mathbf{FI_{NF}}$ ($T_m = 10$)	3.2344	-1215	-1193	0.0122
$\mathbf{P_{NF}}$-$\mathbf{FI_{NF}}$ ($T_m = 20$)	2.7223	-1263	-1241	0.0103
$\mathbf{U_{NF}}$-$\mathbf{I_{NF}}$	0.8419	-1585	-1559	0.0032
$\mathbf{U_{NF}}$-$\mathbf{FI_{NF}}$ ($T_m = 5$)	0.6936	-1638	-1613	0.0026
$\mathbf{U_{NF}}$-$\mathbf{FI_{NF}}$ ($T_m = 10$)	0.6647	-1650	-1625	0.0025
$\mathbf{U_{NF}}$-$\mathbf{FI_{NF}}$ ($T_m = 20$)	0.7932	-1601	-1576	0.003
$\mathbf{U_{NF}}$-$\mathbf{U_{NF}}$	0.2910	-1876	-1847	0.0011
